# HSP superfamily of genes in the malaria vector *Anopheles sinensis*: diversity, phylogenetics and association with pyrethroid resistance

**DOI:** 10.1186/s12936-019-2770-6

**Published:** 2019-04-11

**Authors:** Feng-Ling Si, Liang Qiao, Qi-Yi He, Yong Zhou, Zhen-Tian Yan, Bin Chen

**Affiliations:** 1 School of Life Sciences, Chongqing University, Chongqing, 401331 China; 2 Chongqing Key Laboratory of Vector Insects, Institute of Entomology and Molecular Biology, Chongqing Normal University, Chongqing, 401331 China

**Keywords:** *Anopheles sinensis*, Heat shock protein (HSP), Genome-wide identification, Characteristics, Expression profile, Pyrethroid resistance

## Abstract

**Background:**

Heat shock proteins (HSPs) are molecular chaperones that are involved in many normal cellular processes and various kinds of environmental stress. There is still no report regarding the diversity and phylogenetics research of HSP superfamily of genes at whole genome level in insects, and the HSP gene association with pyrethroid resistance is also not well known. The present study investigated the diversity, classification, scaffold location, characteristics, and phylogenetics of the superfamily of genes in *Anopheles sinensis* genome, and the HSP genes associated with pyrethroid resistance.

**Methods:**

The present study identified the HSP genes in the *An. sinensis* genome, analysed their characteristics, and deduced phylogenetic relationships of all HSPs in *An. sinensis*, *Anopheles gambiae*, *Culex quinquefasciatus* and *Aedes aegypti* by bioinformatic methods. Importantly, the present study screened the HSPs associated with pyrethroid resistance using three field pyrethroid-resistant populations with RNA-seq and RT-qPCR, and looked over the HSP gene expression pattern for the first time in *An. sinensis* on the time-scale post insecticide treatment with RT-qPCR.

**Results:**

There are 72 HSP genes in *An. sinensis* genome, and they are classified into five families and 11 subfamilies based on their molecular weight, homology and phylogenetics. Both RNA-seq and qPCR analysis revealed that the expression of *AsHSP90AB*, *AsHSP70*-*2* and *AsHSP21.7* are significantly upregulated in at least one field pyrethroid-resistant population. Eleven genes are significantly upregulated in different period after pyrethroid exposure. The HSP90, sHSP and HSP70 families are proposed to be involved in pyrethroid stress response based in expression analyses of three field pyrethroid-resistant populations, and expression pattern on the time scale post insecticide treatment. The *AsHSP90AB* gene is proposed to be the essential HSP gene for pyrethroid stress response in *An. sinensis*.

**Conclusions:**

This study provides the information frame for HSP superfamily of genes, and lays an important basis for the better understanding and further research of HSP function in insect adaptability to diverse environments.

**Electronic supplementary material:**

The online version of this article (10.1186/s12936-019-2770-6) contains supplementary material, which is available to authorized users.

## Background

The term “heat shock protein” (HSP) represents a superfamily of genes, and their proteins as “molecular chaperons” increase in synthesis in response of unfavorable environmental condition in both prokaryotic and eukaryotic cells [1]. Most of the HSPs have conserved sequences through bacteria to human, but their expression and function subject to environmental stress do vary from organism to organism [2]. The HSPs are traditionally classified into eight families based on their molecular weight (MW) from 10 to 110 kDa and homology: HSP110 (HSPH), HSP90 (HSPC), HSP70 (HSPA), HSP40 (DNAJ), small HSP (sHSP, HSPB), and the chaperonin families HSPD (HSP60), HSPE (HSP10) and CCT (TRiC) [3]. There have been a number of functional studies on HSPs in bacteria, algae, plant, amphibians, birds, and mammalian, especially in the model organisms of *Anopheles gambiae*, *Drosophila melanogaster*, *Arabidopsis thaliana*, *Saccharomyces cerevisiae*, *Caenorhabditis elegans*, *Danio rerio* and *Mus musculus* [4–7]. The HSP genes of insects encode molecular chaperones that help repair stress injuries via transportation and degradation of aggregated proteins, and they are susceptible to environment stresses, such as heat or cold stress, and insecticidal infection in insects [8, 9]. The HSP expression patterns have been identified in *Drosophila* species and some other insects [10–13]; however, the information regarding HSP whole-genome diversity and association with insecticide resistance is still quite limited for insects as well as mosquitoes.

Recent research showed that *HSP90* was not only an important gene in *Apolygus lucorum* adults in response to extremely high temperature, but was also associated with the resistance or tolerance to cyhalothrin, imidacloprid, chlorpyrifos, and emamectin benzoate and cyhalothrin [14]. Two *HSP70s* were upregulated in a chlorpyrifos-resistant population of *Plutella xylostella*, whereas six *sHSPs* were downregulated [15]. The *sHSPs* expression in responses of indoxacarb and cantharidin varied, and the exposure to beta-cypermethrin and chlorfenapyr resulted in an increase of 13 *sHSP* transcripts and a reduction of 12 *sHSP* transcripts in *Plutella xylostella*, respectively, which indicates that different *sHSPs* might play distinct roles in the development and regulation of physiological activities [16]. One *HSP70* was highly expressed in the captafol-exposed larvae of *Drosophila melanogaster* [17]. In the imidacloprid and deltamethrin treatment groups, only one *HSP90* was upregulated more than two-fold in response to deltamethrin treatment in *Sogatella furcifera* [18]. Two *HSPs* were up-regulated in DDT-resistant field isolates in *An. gambiae*, supporting their link with insecticide resistance and/or stress response [19]. *HSPs* are induced in response to stress induced by environmental factors and may be involved in adverse reactions, including insecticide resistance [20].

Mosquitoes transmit disease resulting in around 700 million people of mosquito-borne illness each year and over one million deaths [21]. Mosquito control relies primarily on the use of insecticides through insecticide-impregnated bed nets and indoor residual spray. In the past decades, pyrethroids have become the preferred insecticide because of their low toxicity to humans, high efficacy against mosquito vectors and short residual action. However, there has been increasing number of mosquitoes to have developed resistance to pyrethroids [22]. *Anopheles sinensis* is a major malaria vector in China and other Southeast Asian countries [23]. Because of extensive and continued application, pyrethroid resistance in *An. sinensis* is now widespread in many regions of China [24, 25]. However, the molecular mechanisms of pyrethroid resistance in *An. sinensis* are not yet clearly understood and pose a challenge for the control of malaria in China. Recently, *An. sinensis* genome was deeply sequenced and assembled, and are comprehensively conducting the research on the molecular mechanism of pyrethroid resistance using the species as model. Some families of genes have been identified at whole-genome level, and their association with pyrethroid resistance have been investigated or summarized, such as cytochromes P450s [26] and carboxylesterases [27]. However, there is still no report for the genome-wide identification of the HSP superfamily of genes in insects as well as in *An. sinensis*. The association of *HSPs* with pyrethroid resistance has been little known in insects as well as in *An. sinensis*.

The present study identified and classified the HSPs superfamily of genes in *An. sinensis* genome, and analysed their phylogenetics and basic characteristics. More importantly, this study screened the HSP genes associated with pyrethroid resistance at whole-genome level through transcription comparison between resistant- and susceptible populations/strain from three geographical regions, quantitative PCR confirmation of candidate HSP genes, and expression changes of HSP genes in response to pyrethroid exposure. This study provides a comprehensive information frame for the HSP superfamily of genes in the *An. sinensis* and other insects, and lays a foundation for the functional study of HSPs in relation of pyrethroid resistance.

## Methods

### Whole-genome identification of *Anopheles sinensis* HSP genes

The HSP genes were identified from the genome and two sets of transcriptome sequences of *An. sinensis*. The former was achieved by Institute of Entomology and Molecular Biology, Chongqing Normal University, China (publication in preparation), and the later were downloaded from the GenBank [24, 28]. Three approaches were used to the identification as previously described for the gene searching at whole-genome level in other species. First, this study searched the *An. sinensis* amino acid (a.a.) database and nucleotide database using BLAST (Basic Local Alignment Search Tool) [29] with E-value cut off of 1 × 10^−5^ using HSP sequences of *Homo sapiens* and *An. gambiae* as query. Second, *An. sinensis* HSP sequences retrieved by five hidden Markov model (HMM) for the HSP superfamily (HSP90, PF00183; HSP70, PF00012; HSP60, PF00118; HSP40, PF01556; HSP20, PF00011) to search against *An. sinensis* a.a. database using Pfam (version 27.0) (http://pfam.sanger.ac.uk/) [30]. Third, this study combined the sequences retrieved from two approaches above as query to search against the *An. sinensis* genome assembly with an E-value cut-off of 1 × 10^−5^. Then putative HSP sequences were predicted by Fgenesh+ (http://www.softberry.com/) to determine whether they are full-length or partial sequences of genes. Full-length sequences of the partial sequences of genes were determined by extending the flanking regions (1 kb or longer), and manually corrected by comparison with other homologous HSP genes. Finally, the HSP genes and their sequences were determined in combining the results from these steps above. The scaffold position of each HSP gene was mapped to the *An. sinensis* genome using BLAST searching, and the scaffold distribution of HSPs was drawn manually. The scaffolds containing only one gene were not displayed, and the gene clusters were defined by the presence of four or more genes in a region of less than 200 kb [31].

### Phylogenetics analysis and characterization of *An. sinensis* HSPs

The deduced HSP a.a. sequences of *An. sinensis*, and the HSP a.a. sequences of *An. gambiae*, *Aedes aegypti*, and *Culex quinquefasciatus* downloaded from NCBI (http://www.ncbi.nlm.nih.gov/database) were used to refer the phylogenetic relationships of HSP genes. The alignment of full amino acid sequence was conducted using ClustalW2.0 program with the default Needle algorithm [32] and corrected manually using GeneDoc [33]. Phylogenetic trees were constructed using the neighbor-joining (NJ) algorithm with the Poisson model, p-distance and pairwise deletion of gaps using MEGA5.0 [34]. The maximum likelihood (ML) method was also used to infer the phylogenetic relationships using MEGA5.0 with the best-fit model (JTT + G+I + F) predicted by the Modeltest 3.7 [34]. Tree topology was assessed by bootstrap analysis with 1000 resampling replicates. The deduced a.a. sequences were further confirmed by the analysis of its characteristics. The theoretical molecular weights and isoelectric points of the predicted a.a. were calculated using the ExpaSy server (http://www.expasy.ch/tools/pi tool.html). The signal peptide sequences were predicted using SignalP 4.1 server (http://www.cbs.dtu.dk/services/SignalP/), and the subcellular localization using the Wolfpsort (http://www.genscript.com/psort/wolf_psort.html). The conserved domains were examined using the program Pfam (version 27.0) (http://pfam.sanger.ac.uk/) [30], and the gene structures were determined from the Gene Structure Display Server (http://gsds.cbi.pku.edu.cn/).

### Screening of HSP genes associated with pyrethroid resistance using RNA-seq

Four populations/strain of *An. sinensis* were used to screen HSP genes associated with pyrethroid resistance using RNA-seq. They are three field pyrethroid-resistant populations from Anhui Province (AH-FR), Chongqing Municipality (CQ-FR), and Yunnan Province (YN-FR), and one laboratory pyrethroid-susceptible strain originally collected from Wuxi, Jiangsu Province (WX-LS). The mosquito larvae and pupae from fields were collected from rice fields, and were reared to adults in an insectary under 27 ± 1 °C and 70 ± 10% RH. The female adults 3 day post emergence, which were preliminarily identified to *An. sinensis* using morphological characters, were tested for pyrethroid susceptibility using the standard WHO tube bioassay using test papers with a diagnostic concentration of deltamethrin (0.05%) (CAS: 52918-63-5, Sigma-Aldrich, St. Louis, MO, USA) [35]. The individuals that were still alive post 24 h of recovery after 1 h 0.05% deltamethrin treatment were considered to be deltamethrin resistant. All individuals in the *An. sinensis* susceptible laboratory strain died in the first 1 h 0.05% deltamethrin treatment. Prior to RNA extraction, genomic DNA was extracted from two or three legs of each individual mosquito for molecular identification using the ribosomal DNA internal transcribed spacer 2 (rDNA-ITS2)-based method to confirm the morphological identification [36].

At least 100 female adults 3 day post emergence from each of these three pyrethroid-resistant populations and the laboratory strain were preserved in RNAlater (Qiagen Shanghai, China) for RNA extraction and RNA-seq library construction performed as previously reported [28], one pool of 15 individuals was used for the RNA-seq, respectively. After RNA quality was assessed using an Agilent 2100 Bioanalyzer (Agilent, Santa Clara, CA), the cDNA for each library was synthesized and amplified using the Mint-2 cDNA synthesis kit (Evrogen, Moscow, Russia). Illumina HiSeq™ 2000 was used for cDNA library sequencing at Beijing Genomics Institute (BGI), following to manufacturer’s instructions. Reads obtained from Illumina sequencing were mapped to the *An. sinensis* genome using TopHat [37], and expression were determined in term of fragment per kb per million reads (FPKM) using Cufflinks [37, 38]. Differential accumulation of transcripts between deltamethrin-resistant and -susceptible mosquitoes was assessed by the Cuffdiff program within Cufflinks. To minimize the impact of sequencing length and nucleotide composition, FPKM for each gene of each sample were calculated to determine the expression quantity [38]. The gene comparison pair (field-resistant/library-susceptible sample) with the FPKM fold change ≥ 2 [39, 40] and the *p* value ≤ 0.05 [− Log10(*p*-value) ≥ 1.301] were considered to significantly up-regulated [41]. In contrast, the pair with FPKM fold change ≤ 2 and the *p*-value ≤ 0.05 ≥ 1.301] were treated as significantly down-regulated [41].

### qPCR verification of potential HSP genes associated with pyrethroid resistance

The genes significantly up-regulated at least one resistant population in the RNA-seq analysis and those genes earlier reported associated with insecticide/deltamethrin resistance were chosen to carry out reverse-transcription quantitative-PCR (RT-qPCR) verification. These three pyrethroid resistant populations and the laboratory susceptible strain were investigated for the expression confirmation of selected genes. The RT-qPCRs were conducted with three biological replicates (three mosquitoes per sample) and three technique replicates. Total RNA was isolated from dehydrated/rehydrated samples using Trizol Reagent (Invitrogen, Shanghai, China) following the supplier’s instructions. RNA concentration was measured using a Nanodrop-1000 spectrophotometer (Thermo scientific). For each sample, complementary DNAs (cDNAs) were synthesized from 1.0 μg RNA, treated with PrimScript ™ RT Reagent Kit with gDNA Eraser (TaKaRa, Dalian, China) and stored at − 20 °C. Gene-specific RT-qPCR primers were designed using Primer Premier 5.0 against the *An. sinensis* genome sequence (Table 1). Real-time reactions were conducted on a thermal cycler (CFX, Bio-Rad, USA) in a 20 μL reaction system containing 10.0 μL of 2 × qPCR mix (Bio-Rad, USA), 0.8 μL each of gene-specific primers (10 mM each) and 1 μL the cDNAs templates, and 7.4 μL of double distilled water. Thermal cycling conditions were 94 °C for 3 min; 40 cycles of 95 °C for 5 s, 60 °C for 15 s, and 72 °C for 15 s, and followed by melting curve analysis (60 to 95 °C). The relative normalized expression was calculated using Bio-Rad CFX96 software. Samples were normalized using the ribosomal protein S7 (*RPS7*) and the ribosomal protein L49 (*RPL49*) Ct values. Basal expression levels were represented as folds over the expression levels of *RPS7* and *RPL49*. Expression folds were calculated with the 2^−∆∆Ct^ method [42] between treatment and control samples for each biological replicate. All data were presented as mean ± SD (standard deviation) of three biological and three technical replicates. Significant differences between treatment groups and the control group were analysed using Student’s t test with *p* ≤ 0.05 pairs. One-way ANOVA was used for multiple comparisons.

**Table 1:** Primers used for qPCR verification for the potential genes associated with pyrethroid resistance, detected by RNA-seq

Genes	Primer names	Primer sequence (5′–3′)	Amplification length (bp)
*AsHSP90AB*	HSP90AB-QF	GAAGATGGACATTATGGACGCAG	161
	HSP90AB-QR	GCGAGATGTCGTCGGCATTG	
*AsHSP90AA*	HSP90AA-QF	CAAGGATGATGAGCCGAAGC	148
	HSP90AA-QR	CAATGCCGACGACATCTCGC	
*AsHSP90B1*	HSP90B1-QF	GAGGGCGAGGTTACGTTCAA	153
	HSP90B1-QR	ACTCGTCCGTGATGAACACC	
*AsTRAP*	TRAP-QF	CGAATCCGGGAGGTAATCCG	121
	TRAP-QR	CCCACGAACCGGTAGAACTC	
*AsHSP70*-*2*	HSP70-2-QF	CCCTCTTTTCTGGCTTCGGA	127
	HSP70-2-QR	AATGGTAGCGTGAGGAAGCC	
*AsDNAJB4*	DNAJB4-QF	TCACACCTCTGCATTGGGTC	136
	DNAJB4-QR	ATCCCCACGAGGAAAGGCTA	
*AsHSP21.7*	HSP21.7-QF	CGAATCCGGGAGGTAATCCG	151
	HSP21.7-QR	CCCACGAACCGGTAGAACTC	
*AsHSP13.7*	HSP13.7-QF	TTCGGACGGTATCCTGACCA	117
	HSP13.7-QR	GCAGACTGTCCGTTCTCCAG	
*AsHSP17.8*	HSP17.8-QF	GGACGAGCATGGCTACATCT	106
	HSP17.8-QR	TCCTCTCTGGGGCAAGTGAT	
*AsHSP21*	HSP21-QF	ATGAACCGCGAGTCCAACTT	128
	HSP21-QR	TGGCGAGCTAATATCGGACG	
*AsHSP23.5*	HSP23.5-QF	ATGTACTTCCGCGACTGGTG	148
	HSP23.5-QR	TACCGAAATCACTCCACGGC	
*AsHSP24.5*	HSP24.5-QF	CAAAATCAGCATCCGGGTCG	125
	HSP24.5-QR	GTGTCGCGAGATGTAGCCAT	
*AsHSP24.7*	HSP24.7-QF	CAGGACGAGCATGGCTACAT	151
	HSP24.7-QR	TCACAGCTTCTTTGGCAGAC	
*AsHSP25.6*	HSP25.6-QF	TCGGACGGTATCCTGACCAT	137
	HSP25.6-QR	TCCATTTTCTCTCCTTCGGGC	
*AsHSP19*	HSP19-QF	TGTTGATGAGCTCTGTGCTG	122
	HSP19-QR	TGGCGAGCTAATATCGGACG	
*AsRPL49*	RPL49-QF	GGAGCCGGTCGGTGATATGT	121
	RPL49-QR	TTCCTTCTCGGTCGGCTTCG	
*AsRPS7*	RPS7-QF	CGGAGAAGATGGCATGGGAGAT	148
	RPS7-QR	ATAGTGAGCATAGGCCCGGTTA	

### Expression analysis of HSP90 and sHSP genes under pyrethroid stress

All four HSP90 family of genes and all ten sHSP family of genes identified in the present study were assessed for the expression response on time-scale subject to pyrethroid treatment, because these two families of genes had more reports to be associated with insecticide resistance. The fourth-instar stage larvae of laboratory pyrethroid-susceptible strain were employed for the assessment. The 50 percent lethal (LC_50_) concentration with deltamethrin was first determined as the pyrethroid treatment concentration of the larval samples. Five pyrethroid concentrations (0.00059, 0.00118, 0.00256, 0.00512, 0.01000 mg/L) were applied for the determination under the standard insectary condition indicated above. For each concentration, 30 larvae without feeding were placed in 200 ml of treated or untreated water in trays with four replicates, larval mortality was recorded 24 h later, and the LC_50_ was calculated based on the mortality using WHO standard method [43]. Thirty-fourth larvae were subsequently treated with the LC_50_ of deltamethrin or untreated water as control for 24 h, and then moved to rearing-conditioned water with four replicates. The samples were collected at 1 h, 3 h, 6 h, 12 h, 24 h, 36 h and 48 h post moving to water, and were subject to expression analysis using qPCR technique with three biological and three technical replications as stated above.

## Results

### HSP superfamily of genes and their classification in *Anopheles sinensis* genome

A total of 72 HSP genes are identified from *An. sinensis* genome and transcriptome database (Additional file 1: Table S1). Among these genes, 71 genes have complete open reading frame (ORF) and are supported by transcripts, and one gene (*AsDNAJB9*) also has complete ORF sequence but no transcript has been found. The deduced a.a. sequence of *AsDNAJB9* shares 46% sequence identity to other reported insect HSP40 s and has the conserved domain for HSP40 recognition; therefore, it is also considered as a true HSP40 gene. These 72 genes were classified into five families (HSP90, HSP70, chaperonins, HSP40 and sHSP) and 11 subfamilies based on the molecular weight and homology analysis. The nomenclature of HSP90 and HSP70 family also refers the nomenclature for *An. gambiae* HSP genes retrieved from VectorBase, and the nomenclature of chaperonins and HSP40 follows the nomenclature of the HUGO Gene Nomenclature Committee. There are 69, 69 and 88 HSP genes in *An. gambiae*, *Cx. quinquefasciatus* and *Aedes aegypti* genomes in the present study, respectively, and they are also classified into five families and 11 subfamilies (Table 2). There are 40, 36, 25 and 35 genes in HSP40 family, 11, 11, 14 and 13 genes in Chaperonins family, 10, 10, 14 and 24 genes in sHSP family, 7, 8, 11 and 8 genes in HSP70 family, and 4, 4, 5 and 8 genes in HSP90 family, separately in *An. sinensis*, *An. gambiae*, *Culex quinquefasciatus* and *Aedes aegypti*. It appears that the HSP40 family is the largest family in term of gene number, and the HSP90 family is the smallest family for all of these four species investigated.

**Table 2:** Family, subfamily and number of the HSP genes in *An. sinensis*, *An. gambiae*, *Cx. quinquefasciatus* and *Ae. aegypti* genomes

Family/subfamily	*An. sinensis*	*An. gambiae*	*Cx. quinquefasciatus*	*Ae. aegypti*
HSP90 family	4	4	5	8
HSP90A subfamily	2	2	3	6
HSP90B subfamily	1	1	1	1
TRAP subfamily	1	1	1	1
HSP70 family	7	8	11	8
HSP70 subfamily	5	6	9	6
HSP110 subfamily	2	2	2	2
Chaperonins family	11	11	14	13
CCT subfamily	9	9	12	11
HSPD subfamily	1	1	1	1
HSPE subfamily	1	1	1	1
DNAJ (HSP40) family	40	36	25	35
DNAJA subfamily	4	4	1	2
DNAJB subfamily	8	7	8	9
DNAJC subfamily	28	25	16	24
sHSP family	10	10	14	24
Total	72	69	69	88

### Gene structure and genomic location of *Anopheles sinensis* HSP genes

**Fig. 1:**
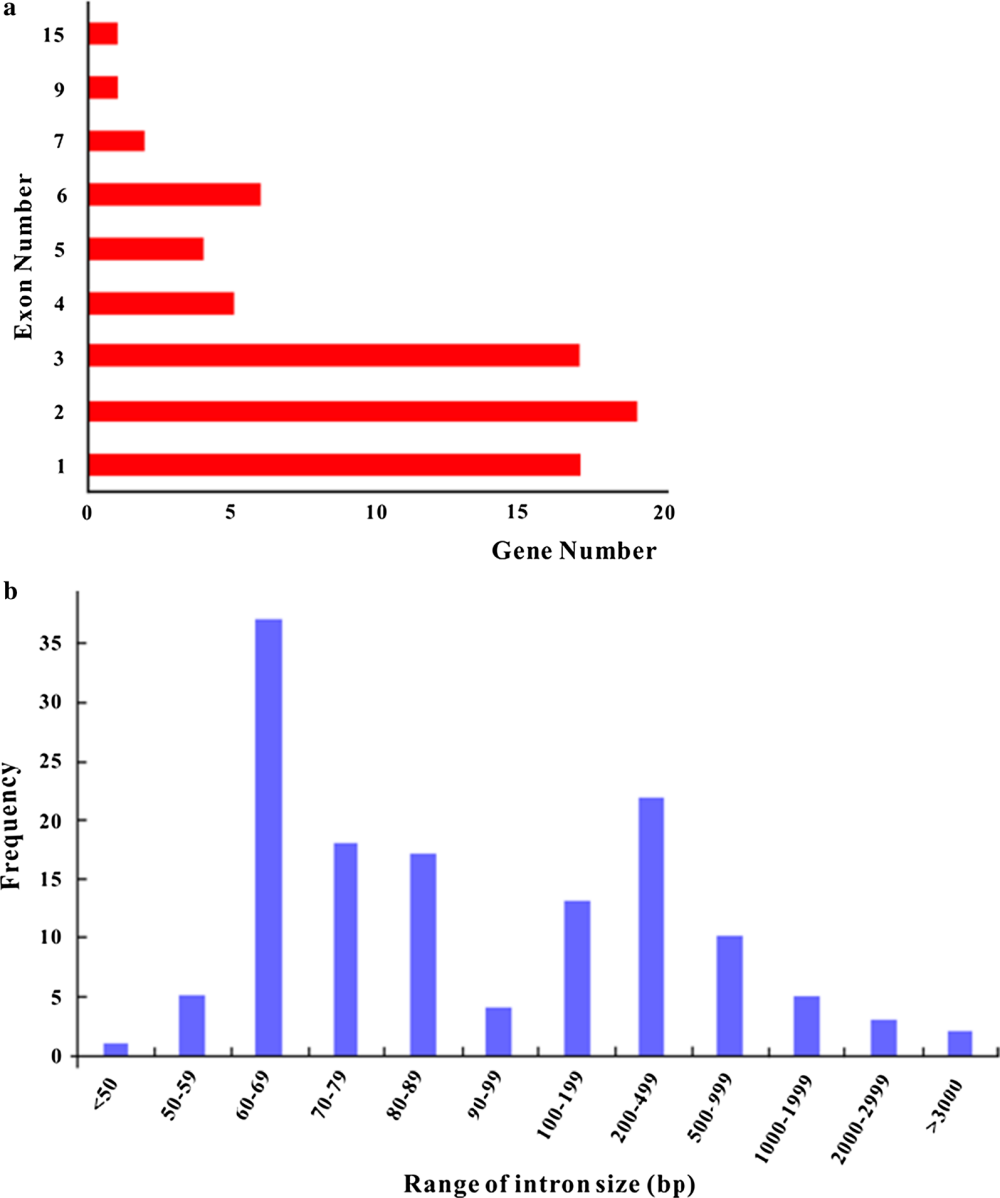
Number/frequency distribution of exon number (**a**) and intron length (**b**) of the 72 HSP genes in *Anopheles sinensis*

The 72 HSP genes have a total of 220 exons with each gene having 1–15 exons, and their exon sizes range from 3 bp in chaperonins family to 2593 bp in HSP70 family (Additional file 1: Table S1). In these genes, 17 genes (23.6% of total) have only one exon (i.e. no intron), 36 genes (50.0%) each have 2–3 exons (i.e. 1–2 introns), 15 genes (20.8%) each have 4–6 exons, and four genes have especially bigger number of exons (7 for HSPH1 and DNAJC23, nine for DNAJC20, and 15 for DNAJC13) (Fig. 1a, Additional file 2: Fig S1). Only HSP40 family has genes with exon number larger than five (except for *HSPH1* in HSP70 family), which might be due to large diversity in the family (40 genes, 55.6% of total). The 17 genes with only one exon exist in the HSP90 (1 gene), HSP70 (1), chaperonins (2), HSP40 (9) and sHSP (4) family, respectively. There are a total 148 introns in the 72 HSP genes, with sizes ranging from 19 to 4790 bp and 37.4% intron having a length of 60–69 bp (Fig. 1b).

**Fig. 2:**
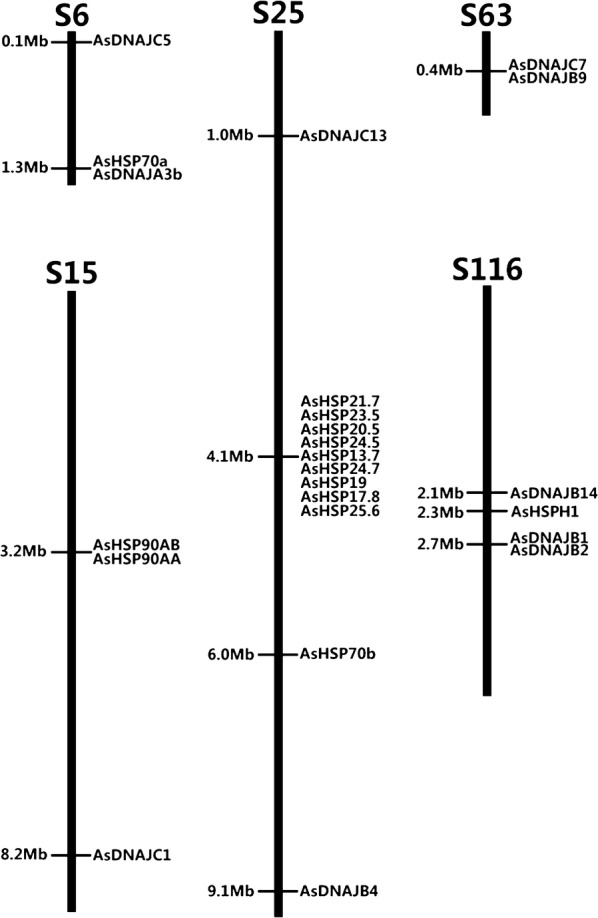
The location of *Anopheles sinensis* HSP genes on Scaffolds. The left side on scaffold (S) denotes physical locations, and right side is for gene names. One individual gene cluster is shown in Scaffolds25

The 72 HSP genes are located on 33 different scaffolds, in which 24 genes are on scaffold6, scaffold15, scaffold25, scaffold63 and scaffold116 (Fig. 2), and 68 genes scattered on 28 other scaffolds with intergenic distance larger than 200 kb. Nine genes in sHSP family are tandemly located in one cluster on scaffold25 with intergenic distance less than 200 Kb, which suggests that these nine genes might be derived from a single gene through a series of gene duplication events. Similarly, *AsHSP90AA* and *AsHSP90AB* in HSP 90 family on scaffold15, *AsDNAJC5* and *AsDNAJB9* in HSP40 family on scaffold63, and *AsDNAJB1* and *AsDNAJB2* in HSP40 family on scaffold116 each cluster together with intergenic distance less than 200 Kb, which suggests that each of these three pairs might experience one gene duplication event. The results reveal that the gene expansion in *An. sinensis* mainly happened in the sHSP family for the function that needs to be further investigated. Most of sHSP genes are also located on one chromosome (chromosome 2) of *An. gambiae* (6 of 10 genes) [47]. The genome of *An. sinensis* is comparative with *An. gambiae* in gene location and possible gene expansion in the sHSP family. The gene expansions in other families need to be further investigated with more insect species.

### Phylogenetic relationships of *Anopheles sinensis* HSP genes

**Fig. 3:**
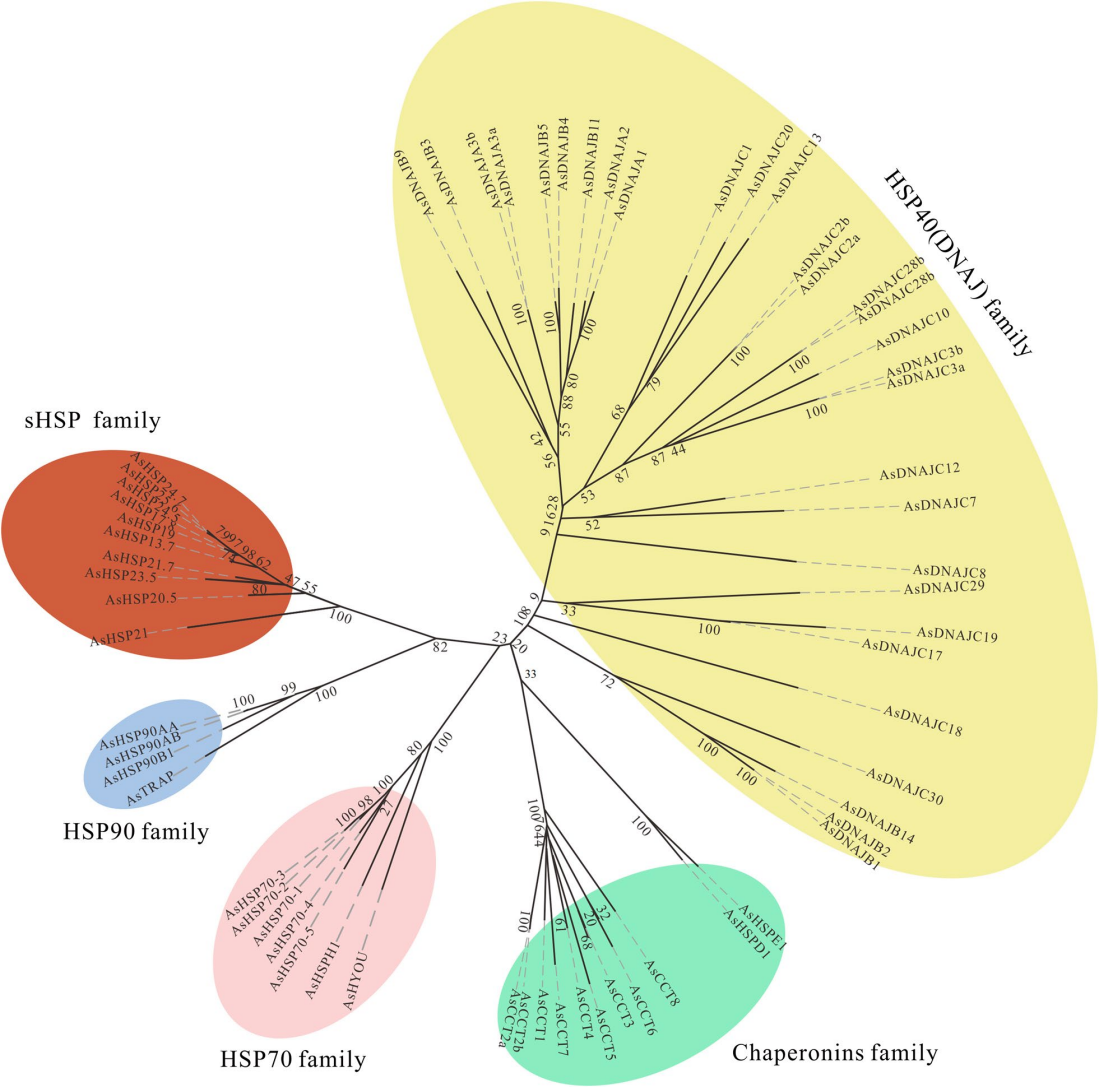
Phylogeny of the HSP genes in *Anopheles sinensi*s. The phylogenetic relationships were inferred based on the amino acids with neighbor-joining method using MEGA5.0. The bootstrap values calculated from 1000 replicates were marked on each corresponding node. HSP90, HSP70, Chaperonins, HSP40 (DNAJ) and sHSP represent five different families in the HSP superfamily

The phylogenetic analysis with NJ method generally classifies the 72 HSP genes in *An. sinensis* into five families as expected: HSP90, HSP70, Chaperonins, HSP40 and sHSP (Fig. 3). The HSP90, HSP70 and sHSP families of clades are each supported by a 100% bootstrap values, which suggests that these three families each be a monophyly; however, the Chaperonins and HSP40 families of branches are only supported by 33% and 20% bootstrap values, respectively, which suggests that these two families be not monophyly. The phylogenetic relationships well fit the modern classification at family level [3]. This study further investigated and discussed the phylogenetic relationships of each family of genes with inclusion of the HSP genes in *An. gambiae*, *Culex quinquefasciatus* and *Aedes aegypti* using amino acid sequences with ML method.

#### HSP90 family (Additional file 3: Fig S2A)

Twenty-one HSP90 genes from *An. sinensis* (4 genes), *An. gambiae* (4), *Culex quinquefasciatus* (5) and *Aedes aegypti* (8) are grouped into three subfamilies, HSP90A (13 genes), HSP90B (4) and TRAP (4). All these three subfamilies are supported by 100% bootstrap value, which suggests that these all be monophyly.

#### HSP70 family (Additional file 3: Fig S2B)

The HSP70 family of genes are currently classified into two subfamilies, HSP70 and HSP110 [3], and these genes function in cytoplasm, mitochondrion and endoplasmic reticulum (ER) [50]. The present research based 34 HSP70 genes suggests that the HSP70 subfamily be a monophyly with 100% bootstrap value, whereas the HSP110 subfamily be a paraphyly. The HSP70 subfamily of genes are further divided into three groups based on their functional sites, cytoplasm, mitochondrion and ER group, which are all supported by 100% bootstrap value. Similarly, the HSP110 subfamily of genes are further divided into cytoplasm and ER group, which are both supported by 100% bootstrap value.

#### Chaperonins family (Additional file 3: Fig S2C)

The chaperonins family of genes are nowadays classified into three subfamilies, CCT, HSPD and HSPE [3]. This research based on 49 genes suggests that the CCT, HSPD and HSPE subfamilies be monophyly, supported by 94%, 100% and 100% bootstrap values, respectively. The CCT subfamily of genes is further divided into eight groups (A to H), which are all supported at least 96% bootstrap value.

#### HSP40 (DNAJ) family (Additional file 3: Fig S2D)

The HSP40 family of genes is used to be classified into three subfamilies, DNAJA, DNAJB and DNAJC [3]. The domain structure of all HSP40 genes identified in *An. sinensis* (Additional file 4: Table S2). The present study based on 136 genes suggest that the DNAJA, DNAJB and DNAJC be monophyly supported by 83%, 99% and 100% bootstrap values, respectively.

#### sHSP gene family (Additional file 3: Fig S2E)

A total of 58 sHSP genes are classified into eight clusters in the phylogenetic analysis, four orthologous clusters and four species-specific clusters. There are 1–2 genes in each of these four orthologous clusters for the four mosquito species investigated. For the four species-specific clusters, there are six, four, ten and ten genes in *An. sinensis*, *An. gambiae*, *Culex quinquefasciatus* and *Aedes aegypti*, respectively.

### HSP genes associated with pyrethroid resistance

**Fig. 4:**
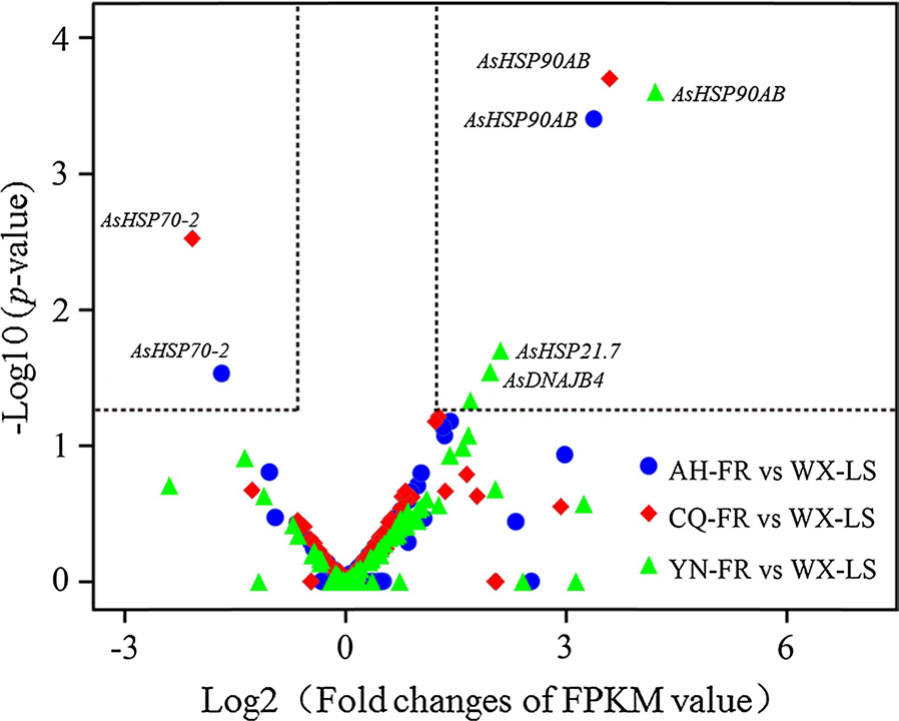
Expression profile of HSP genes in three pyrethroid-resistant populations compared to pyrethroid-susceptible strain (WX-LS) *Anopheles sinensis*, detected by RNA-seq. The three resistant populations are Anhui (AH-FR, marked in blue), Chongqing (CQ-FR, red) and Yunnan (YN-FR, green), respectively. Genes with FPKM value ≥ 2 and *p*-value < 0.05 are considered to be significantly upregulated, whereas those with FPKM value < 0.5 and *p*-value < 0.05 are regarded as significantly downregulated. Vertical dotted lines illustrate the 1 (FPKM = 2) and − 1 (FPKM = 0.5) of Log2 (fold changes of FPKM value) values on the X-axis, and horizontal dotted lines denote the *p*-value = 0.05 of − Log10 (*p*-value) on the Y-axis. *AsHSP90AB* is significantly upregulated in all three resistant populations, *AsHSP70*-*2* is significantly upregulated in both AH-FR and CQ-FR and *AsHSP21.7* and *AsDNAJB4* are significantly upregulated only in YN-FR

Transcript accumulation levels in a RNA-seq experiment are expected to reflect gene transcription. Comparing each of these three field pyrethroid-resistant populations against the laboratory susceptible strain (WX-LS), four HSP genes (*AsHSP90AB*, *AsHSP70*-*2*, *AsHSP21.7* and *AsDNAJB4*) are significantly overexpressed in at least one population with p-values ≤ 0.05 in T-test (Fig. 4). The *AsHSP90AB* is significantly upregulated in all three populations with the folds of 1.58 (in AH-FR), 3.63 (CQ-FR) and 3.74 (YN-FR), the *AsHSP70*-*2* significantly upregulated in both AH-FR (2.56 folds) and YN-FR (3.56), and the *AsHSP21.7* and *AsDNAJB4* alone in YN-FR with the folds of 1.77 and 1.55, respectively. All of the four HSP genes are selected to perform RT-qPCR for validation using the samples consistent with those for RNA-seq analysis.

**Fig. 5:**
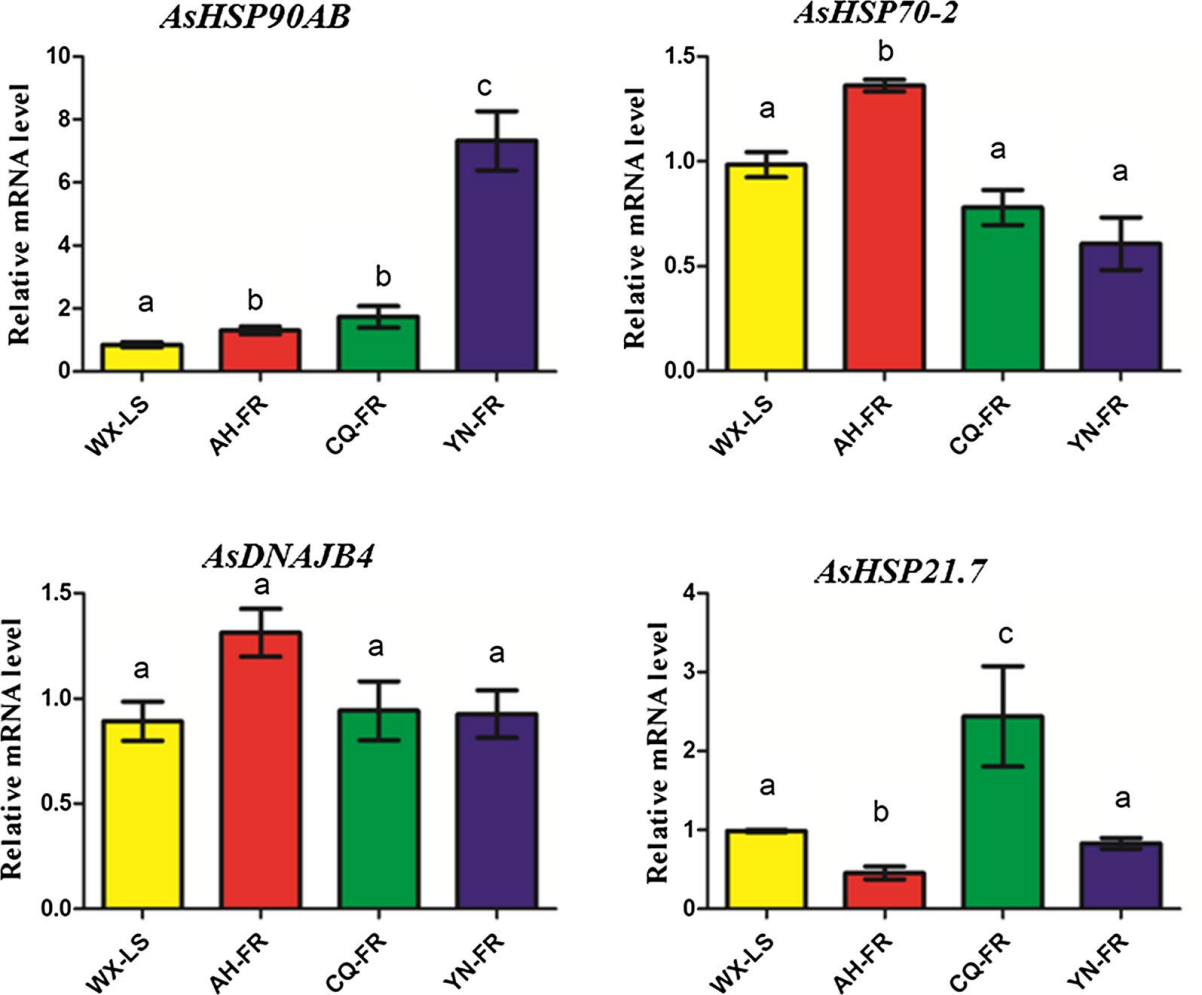
qPCR verification of four genes significantly differentially expressed in RNA-seq analysis. The relative expression levels of these genes are shown as the mean ± SD of three biological and three technical replicates in qPCR analysis. The *RPS7* and *RPL49* genes were used as internal reference for expression normalization. The population/strain pairs marked as different letters are significantly different in expression (*p*-value ≤ 0.05), and those marked as same letters are not (*p*-value ≥ 0.05), determined by one-way ANOVA analysis

The RT-qPCR verification results are largely consistent with those from RNA-seq, and some differences might stem from the different batch of samples, target gene splicing and/or method difference (Fig. 5). Most importantly, the *AsHSP90AB* in HSP90 family is also significantly up-regulated in all three resistant populations in the RT-qPCR analysis. The *AsHSP70*-*2* is significantly up-regulated also in AH-FR but not in YN-FR in the RT-qPCR verification. The *AsHSP21.7* in sHSP family is significantly upregulated in CQ-FR but not in YN-FR in the RT-qPCR verification. The *AsDNAJB4* in HSP40 family has no significant expression difference for any of the three resistant populations in the RT-qPCR verification in comparison of the susceptible strain.

### Expression response of HSP90 and sHSP genes under pyrethroid stresses

**Fig. 6:**
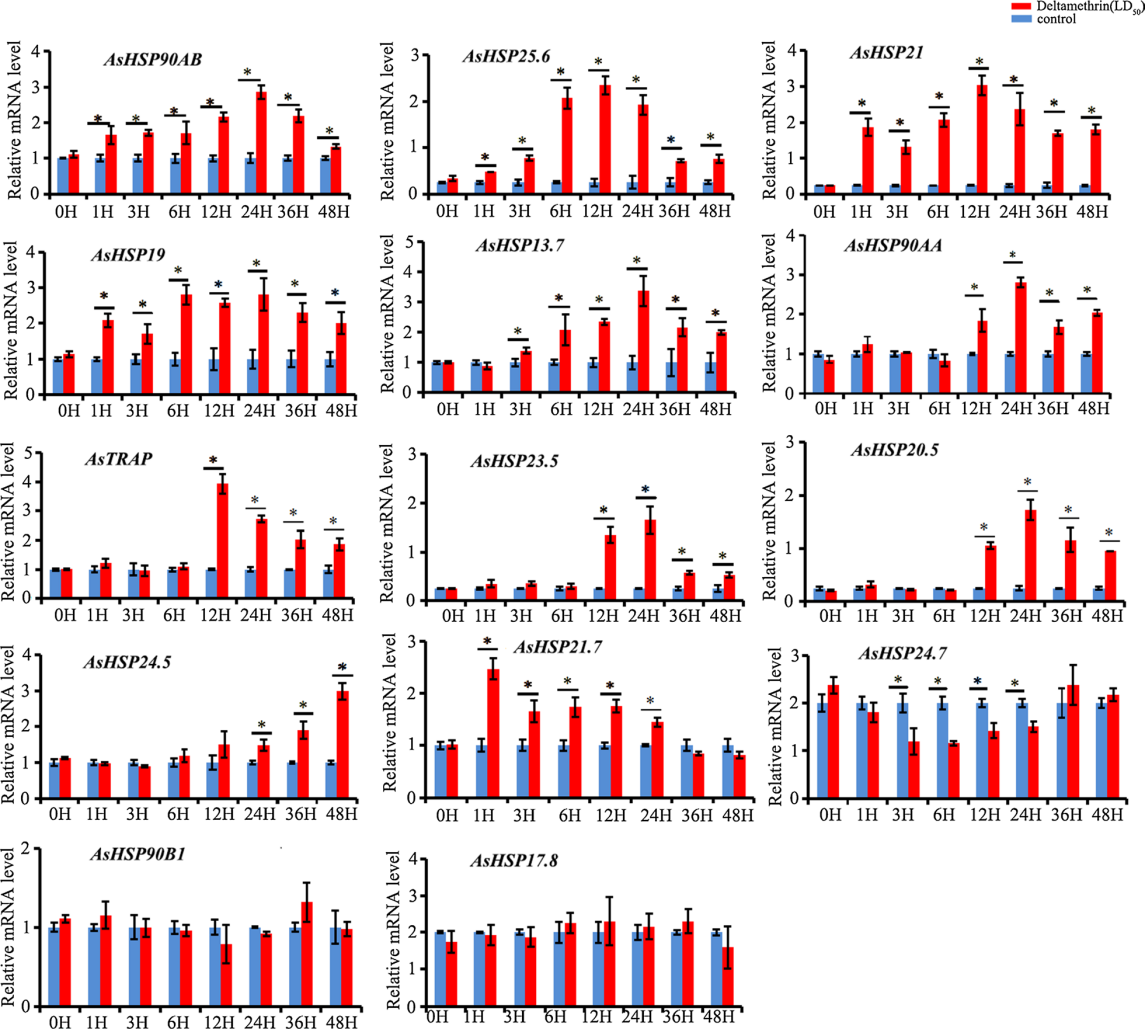
Expression of 14 HSP genes subject to pyrethroid exposure, detected by qPCR. The 14 genes include four from HSP90 family and ten from sHSP family. The fourth-instar larvae at 1 h, 3 h, 6 h, 12 h, 24 h, 36 h and 48 h post pyrethroid treatment were investigated using qPCR analysis with three biological and three technical replications. The expression levels (mean ± SD) of each gene, represented with bars, are normalized in reference of *RPL49* and *RPS7*. The samples marked with “*” were significantly differently expressed (*p*-value ≤ 0.05) compared with the samples without pyrethroid treatment at the corresponding time, and the samples without “*” were not significantly different in expression determined by one-way ANOVA analysis (*p* ≤ 0.05)

The fourth-instar stage larvae of laboratory pyrethroid-susceptible strain were treated with 0.00160 mg/L of deltamethrin, based on the determination of LC_50_ concentration. The obvious expression pattern on the time scale post pyrethroid treatment was detected for the four HSP90 genes and ten sHSP genes. The *AsHSP90AB*, *AsHSP25.6*, *AsHSP19* and *AsHSP21* are significantly up-regulated by 1.33 to 9.47 folds through 1 h to 48 h pyrethroid treatment (Fig. 6); the *AsHSP21.7* is significantly upregulated by 1.44 to 2.47 folds through 1 h to 24 h; the *AsHSP13.7* is significantly upregulated by 1.99 to 3.38 folds through 6 h to 48 h; the *AsHSP90AA*, *AsTRAP*, *AsHSP23.5* and *AsHSP20.5* are significantly upregulated by 1.67 to 6.90 folds through 12 h to 48 h; and the *AsHSP24.5* is significantly upregulated by 1.48 to 2.99 folds through 24 h to 48 h. However, the *AsHSP24.7* is significantly downregulated through 3 h to 24 h, and the *AsHSP90B1* and *AsHSP17.8* show no significant expression variations through 1 h to 48 h. This is the first time to look over the HSP gene expression pattern on the time scale post-insecticide treatment, and the study suggests that both HSP90 and sHSP families genes be overtranscribed across the detoxification process after pyrethroid exposure with specific expression pattern. There is a need to further investigate the involvement of other HSP families of genes.

## Discussion

In history, the HSP superfamily of genes across all organisms was classified into several families based on their molecular weights, HSP105/110, HSP90, HSP70, HSP60, HSP40, small HSP (sHSP) and HSP10 [44, 45]. This is the first report for whole-genome identification of HSP superfamily of genes in the four species as well in insects. However, the HSP110 family does not exist in insect [45], and the HSP10 (HSPE) and HSP60 (HSPD) families have been degraded as subfamilies in insects [46]. The HSP gene number (72) of *An. sinensis* is comparative with that of *An. gambiae* (69) and *Culex quinquefasciatus* (69), but much less than that of *Aedes aegypti* (88). HSPs have been shown to be markedly associated with the resistance to heavy metals, pesticides and oxidative stress in insects, and the difference of gene number might result from the adaptation to different environment [40, 46]. The much larger number in *Aedes aegypti* appears due to the expansion of sHSP family (24 genes in *Aedes aegypti*, and 10–14 in other three species) and HSP90 family (8 genes in *Aedes aegypti*, and 4–5 in other three species).

The established phylogenetic relationship of HSP90 family genes are consistent with earlier studies, in which the HSP90A is a sister with the HSP90B, and the TRAP originated earlier than both HSP90A and HSP90B [48, 49, 69]. Both the HSP90A and HSP90B exist in all eukaryotic kingdoms, and the TRAP also in bacteria [49]. In this study, the four mosquito species investigated are lack of HTPG and HSP90C subfamilies of genes, like in *Drosophila melanogaster* [49]. In the HSP70 family, the C-terminal motif contains with diverse subcellular localizations signatures [50]. In HSP110 subfamily have similar domains as canonical HSP70 subfamily but have long insertions and C-terminal extensions [50]. The ATPase domain and the C-terminal helical lid of HSP110 subfamily were thought to mediate the interaction with HSP70 subfamily [50]. In the chaperonins family, the CCT subfamily of genes in cytoplasm is a multi-subunit protein complex which functions in cytoskeletal protein folding in all eukaryotes [51], and the HSPD and HSPE subfamilies in the mitochondria are orthologs of the *Escherichia coli* GroEL (HSP60) and GroES (HSP10), respectively [3]. It has been reported that HSPE (HSP10) proteins serve as the co-factor of HSPD (HSP60) to assist in the folding of newly synthesized proteins imported into mitochondria [3]. In the HSP40 family, classified into three subfamilies, DNAJA, DNAJB and DNAJC are in accordance with those of previous studies, *Bombyx mori* [52]. For sHSP family, four orthologous clusters and four species-specific clusters was found. This clustering pattern is similar to those in *Bombyx mori* and *An. gambiae* [47].

Previous studies showed that HSP superfamily members were differently expressed in diverse insecticides of insects. This may be correlated with the facts that HSP genes were significant in response to insecticides resistance of insects. This study checked the expression profiles of HSP genes in three field pyrethroid-resistant populations against the laboratory susceptible strain of *An. sinensis*, and the expression patterns of HSP genes verified by qPCR. Similarly, the gene has been reported to be significantly overexpressed in chlorpyrifos-resistant resistance strains in *Plutella xylostella* (HSP90) [53], and in response of DDE induces in *Ruditapes decussatus* (HSP90) [54] and abamectin treatment in *Tetranychus cinnabarinus* (HSP90) [65]. In addition, the increased expression of HSP90 has been associated with pesticide exposure in *Apis mellifera* [55]. The transcriptional expression profile results also indicated that the expression of *AlHSP90* in female adults treated with chlorpyrifos and emamectin benzoate and in male adults treated with cyhalothrin were higher than that with other treatments [14]. Earlier studies show that one *HSP70* gene was significantly upregulated in the DDT-resistant strains of *Aedes aegypti* [56], two *HSP70s* in a chlorpyrifos-resistant population of *Plutella xylostella* [15], one *HSP70* in organophosphorus insecticide resistant *Chironomus yoshimatsu* [20], and two *HSP70s* in chlorpyrifos-resistant *Laodelphax striatella* [57]. One *HSP70* gene has been reported to involve in cellular damage in reproductive tissues induced by cypermethrin insecticide in *Drosophila melanogaster* [58], and Colorado potato beetles produce more HSP70 in response of imidacloprid [59]. The *AsHSP70*-*2* is significantly up-regulated in AH-FR, and a number of HSP70 family of genes were significantly upregulated in populations/strains in a number of species. These findings suggest that the HSP70 families of genes might be also involved in stress response process. These results suggest that HSP70 expression be a sensitive indicator of exposure to certain insecticides and in conjunction with other biomarkers, and it may be useful for assessing exposure to environmental stressors ecosystems [20]. However, HSP70 genes involved might be different along species, geographical populations and insecticides. It is significantly upregulated in response of induce of cypermethrin insecticide in *Caenorhabditis elegans* (*HSP16*) [60]. The expression levels of *sHSP19.7* and *sHSP20.7* in cultured cells of *Mamestra brassicae* were significantly up-regulated in response to high concentrations of chlorfenapyr [61]. However, six *sHSPs* were downregulated in a chlorpyrifos-resistant population of *Plutella xylostella* [15], and one sHSP was downregulated in response of imidacloprid treatment of *Sogatella furcifera* [18]. It appears that some sHSP genes are responsible for the defense against insecticide stress, but the gene response vary upon insecticide and insect species. The *AccDnaJB12* was upregulated from 1 to 1.5 h in response of lambda-cyhalothrin and paraquat treatment in *Apis cerana cerana* [62]. The *OcHsp40* mRNA levels had no significant difference observed with Cd concentrations of *Oxya chinensis* [63].

In arthropods, HSP90 proteins have been shown to be involved in tolerance and resistance to pesticides [64, 65]. HSP90 family of genes has been known to play a role in protein folding and posttranslational regulation, in particular in steroid hormone targeting and cell death and apoptosis regulating [66]. This study reveals that the *AsHSP90AB* is significantly upregulated in the all three pyrethroid-resistant populations investigated and through 1 h to 48 h post pyrethroid exposure, and this result suggests that the *AsHSP90AB* be the essential HSP gene for pyrethroid stress response. The *AsHSP90AA* and *AsTRAP* in HSP90 family might also be involved in pyrethroid stress response. However, three *HSP90* genes and two sHSP genes down-regulated during permethrin exposure on *Anopheles stephensi* third instar larvae [70]. The *AsHSP21.7* is significantly upregulated in CQ-FR, and a number of sHSP family of genes are significantly over-expressed over the detoxification process post pyrethroid exposure. Twelve of 14 sHSPs genes were significantly up-regulated in the fourth instar larvae of *Plutella xylostella* after beta-cypermethrin exposure [15]. These findings suggest that the sHSPs families of genes might be also associated with insecticide stress process. HSP90 and sHSP families genes expression patterns were different in different insecticides and different concentration in different insects.

HSPs are highly demanded by the insects to combat environmental stresses, and are associated with excess expressions of apoptotic genes under insecticide stress, which results in higher apoptosis [53]. In the presence of abiotic and biotic stressors, HSPs upregulated are thought to participate in stress tolerance and promote cell survival mainly through refolding proteins and preventing their denaturation [67, 68]. The difference in expression pattern of these HSPs may be due to a compensation effect among the *HSP* genes [53]. The insect HSPs response to insecticide stress has received increasing attention [49, 53, 62]. In addition, inducible HSPs as playing a vital role in the insecticide resistance phenotype [9]. Previous studies showed that blood feeding increases HSP expression in mosquito, that may suggest a role for HSPs in blood meal-induced reduction of insecticide toxicity [4]. As molecular chaperones, HSPs may be induced by insecticides, and they also contribute to insecticide resistance [20].

## Conclusions

This is the first study to look over the whole HSP superfamily of genes in insects. The study provides useful insights into the diversity, classification, scaffold location, characteristics, and phylogenetics of HSP superfamily of genes in *An. sinensis* genome, and the HSP genes associated with pyrethroid resistance. There are a total of 72 HSP genes in *An. sinensis*, and they are classified into five families (HSP90, HSP70, Chaperonins, HSP40 and sHSP) and 11 subfamilies based on the molecular weight, homology and phylogenetics analyses. The HSP90, sHSPs and HSP70 families of genes are proposed to be involved in pyrethroid stress response based on expression analyses of three field pyrethroid-resistant populations, and expression pattern on the time scale post insecticide treatment. The *AsHSP90AB* is proposed to be the essential HSP gene for pyrethroid stress response in *An. sinensis*. This study provides the information frame for HSP superfamily of genes, and lays an important basis for the better understanding and further research of HSP function in adaptability of insects to diverse environments.

## Additional files

**Additional file 1: Table S1.** Detailed information and intron–exon organization of *An. sinensis* HSPs genes.

**Additional file 2: Fig. S1.** Gene structure of predicted *Anopheles sinensis* HSP genes. Blue boxes represent exons and black lines represent introns. The numbers indicate the splicing phases of the HSP transporter genes: 0 refers to phase 0; 1 to phase 1; and 2 to phase 2.

**Additional file 3: Fig. S2.** Phylogenetic relationships of HSP genes from four insect species. The phylogenetic tree was constructed using the maximum-likelihood method based on predicted amino acid sequences. Analysis was performed with the program package MEGA5.0. The number at the branch point of the node represents the bootstrap values calculated from 1000 replications, and the gaps were deleted with pairwise deletion method. As, *An. sinensis*; Ag, *An. gambiae*; Cq, *Cx. quinquefasciatus* and Ae, *Ae. aegypti*. (A) HSP90 family; (B) HSP70 family; (C) Chaperonins family; (D) HSP40 family; (E) sHSP family.

**Additional file 4: Table S2.** Type and domain structure of 40 HSP40 (DNAJ) genes identified on *An. sinensis* genome. All these genes have complete open reading frame (ORF) sequences, with all supported by transcripts identified.

## Abbreviations

HSPs: heat shock proteins
a.a.: amino acid
ML: maximum likelihood method
NJ: neighbour joining
FPKM: fragment per kb per million reads
HSPH: heat shock protein 110
HSPC: heat shock protein 90
HSPA: heat shock protein 70
CCT: chaperonin-containing tailless complex polypeptide 1
HSPD: heat shock protein 60
HSPE: heat shock protein 10
HMM: Hidden Markov model
WHO: World Health Organization
