# Supplementary figures and images for: HSP superfamily of genes in the malaria vector Anopheles sinensis: diversity, phylogenetics and association with pyrethroid resistance
Source: Malar J. 2019 Apr 11;18:132. doi: 10.1186/s12936-019-2770-6 (PMC6460852; doi:10.1186/s12936-019-2770-6)

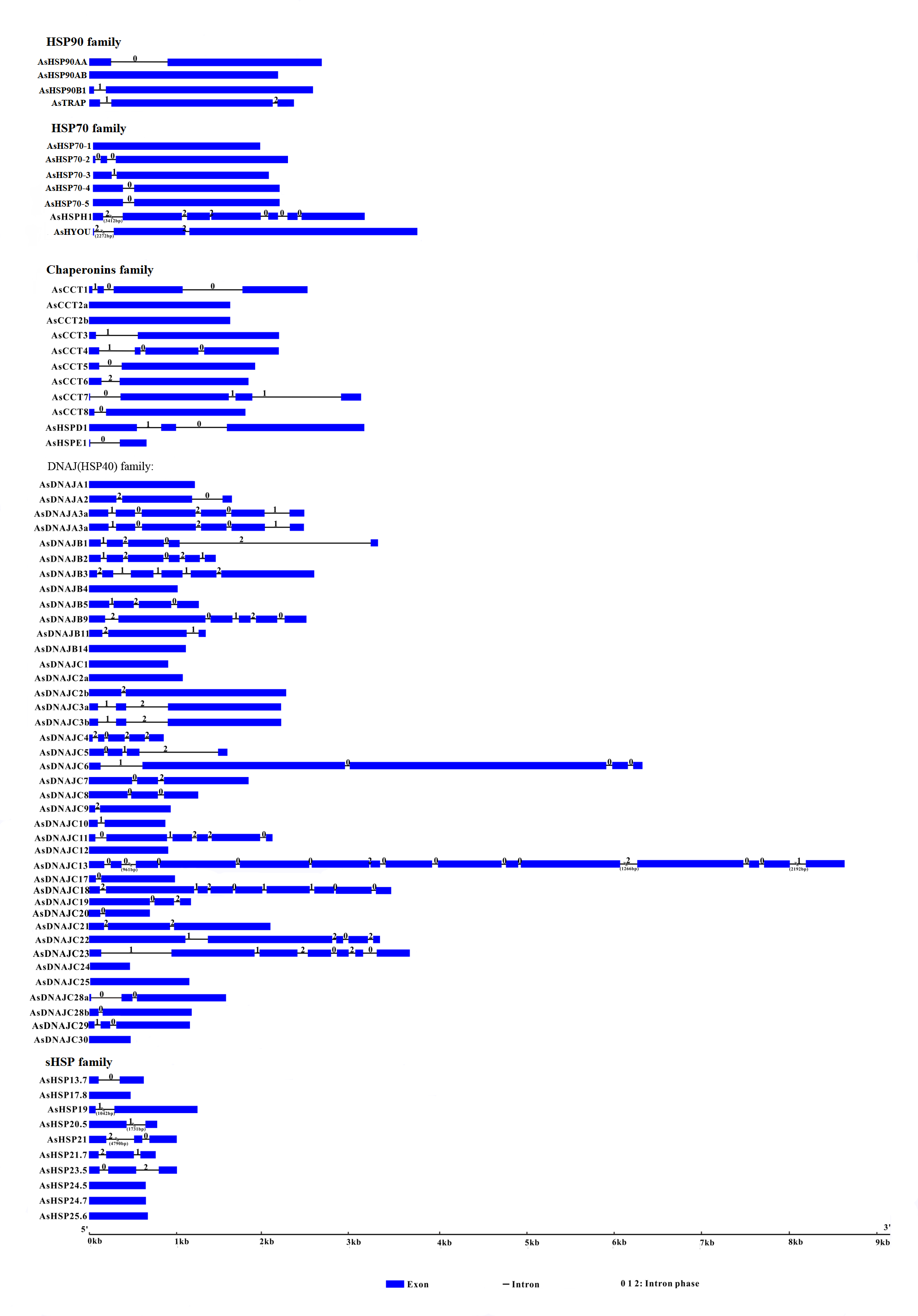

Supplement: Supplementary file 2 — Additional file 2: Fig. S1. Gene structure of predicted Anopheles sinensis HSP genes. Blue boxes represent exons and black lines represent introns. The numbers indicate the splicing phases of the HSP transporter genes: 0 refers to phase 0; 1 to phase 1; and 2 to phase 2. [file 12936_2019_2770_MOESM2_ESM.tif]

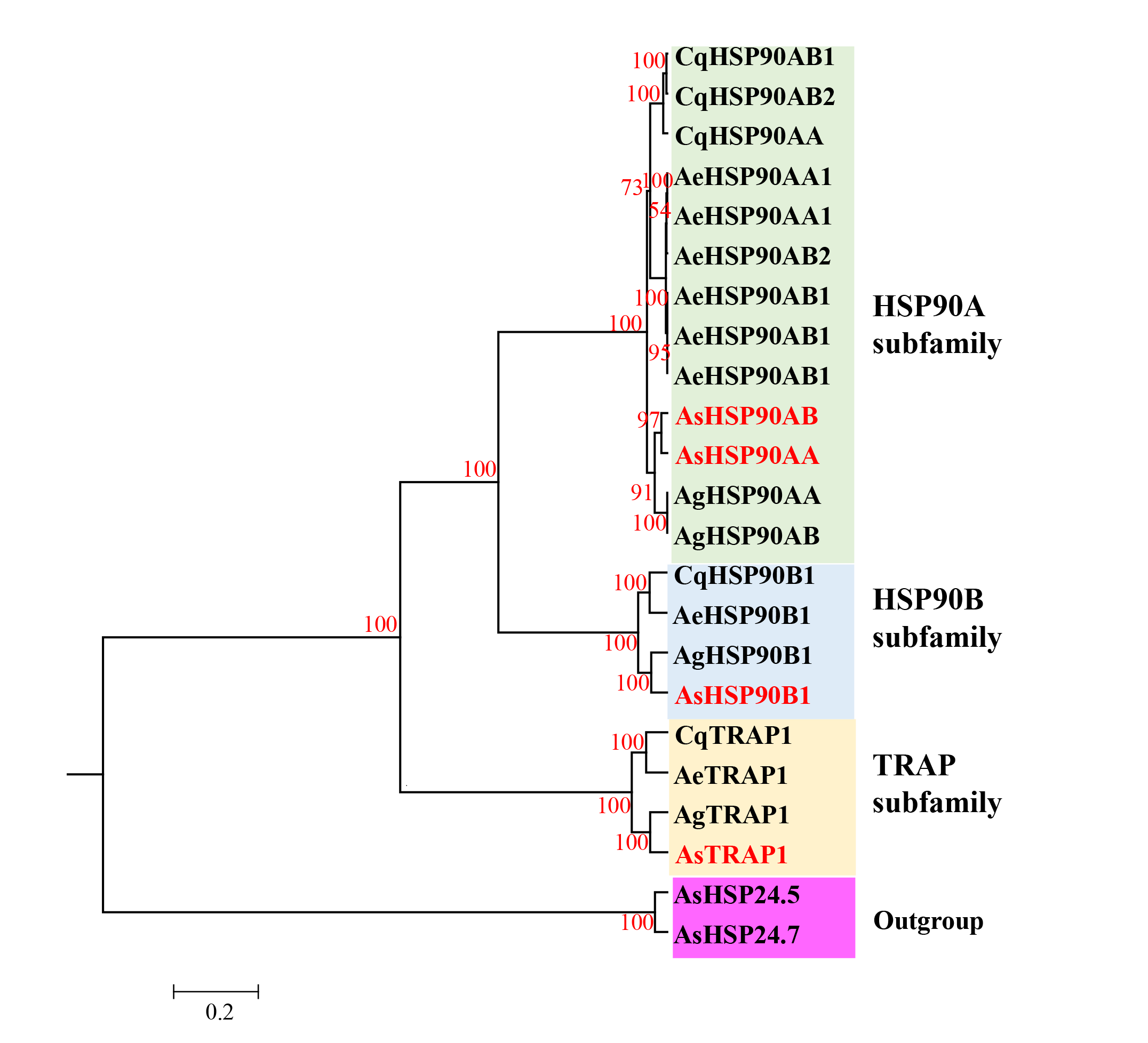

Supplement: Supplementary file 3 — Additional file 3: Fig. S2. Phylogenetic relationships of HSP genes from four insect species. The phylogenetic tree was constructed using the maximum-likelihood method based on predicted amino acid sequences. Analysis was performed with the program package MEGA5.0. The number at the branch point of the node represents the bootstrap values calculated from 1000 replications, and the gaps were deleted with pairwise deletion method. As, An. sinensis; Ag, An. gambiae; Cq, Cx. quinquefasciatus and Ae, Ae. aegypti. (A) HSP90 family; (B) HSP70 family; (C) Chaperonins family; (D) HSP40 family; (E) sHSP family. [file 12936_2019_2770_MOESM3_ESM.zip › Fig S2A.tif]

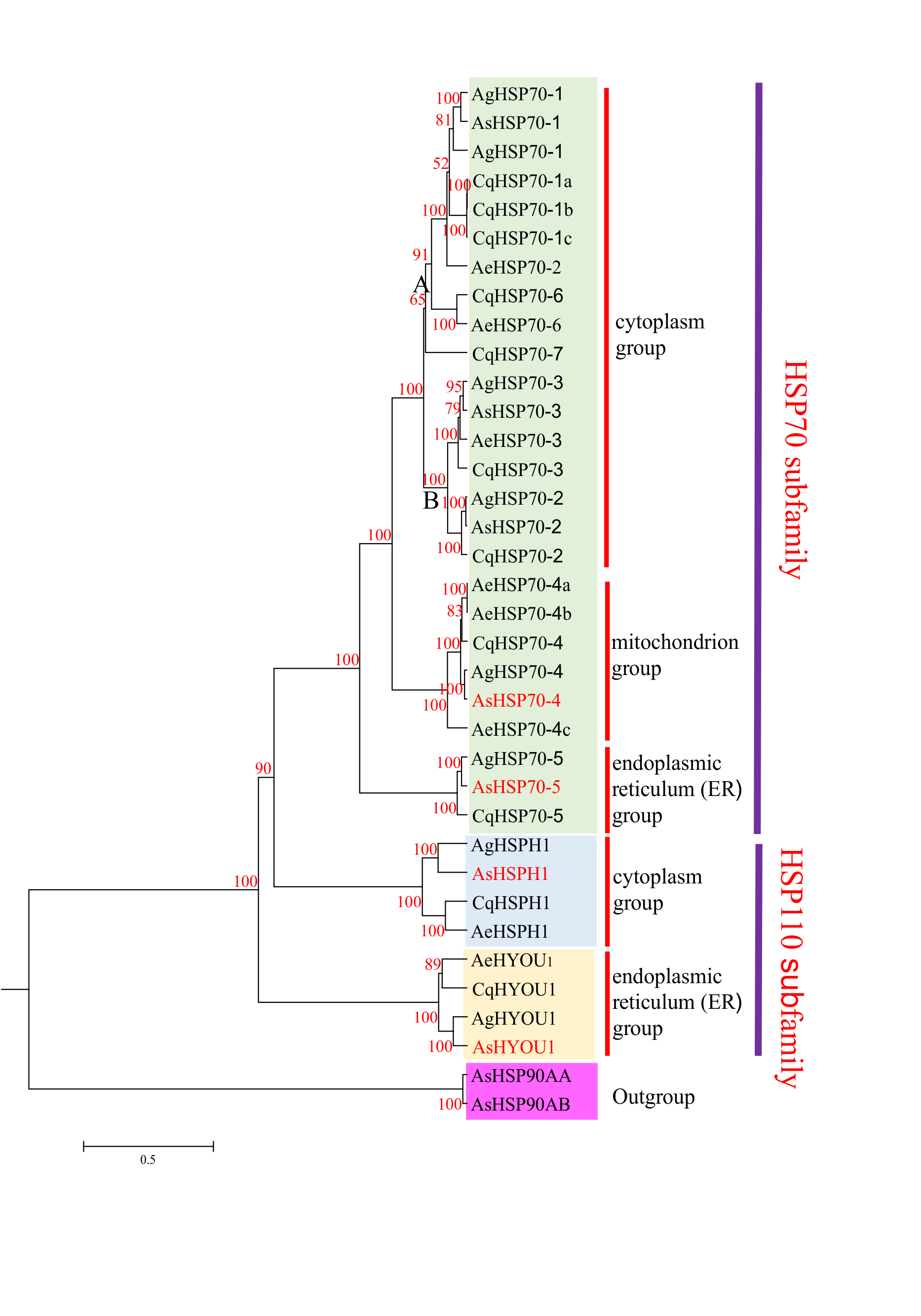

Supplement: Supplementary file 3 — Additional file 3: Fig. S2. Phylogenetic relationships of HSP genes from four insect species. The phylogenetic tree was constructed using the maximum-likelihood method based on predicted amino acid sequences. Analysis was performed with the program package MEGA5.0. The number at the branch point of the node represents the bootstrap values calculated from 1000 replications, and the gaps were deleted with pairwise deletion method. As, An. sinensis; Ag, An. gambiae; Cq, Cx. quinquefasciatus and Ae, Ae. aegypti. (A) HSP90 family; (B) HSP70 family; (C) Chaperonins family; (D) HSP40 family; (E) sHSP family. [file 12936_2019_2770_MOESM3_ESM.zip › Fig S2B.tif]

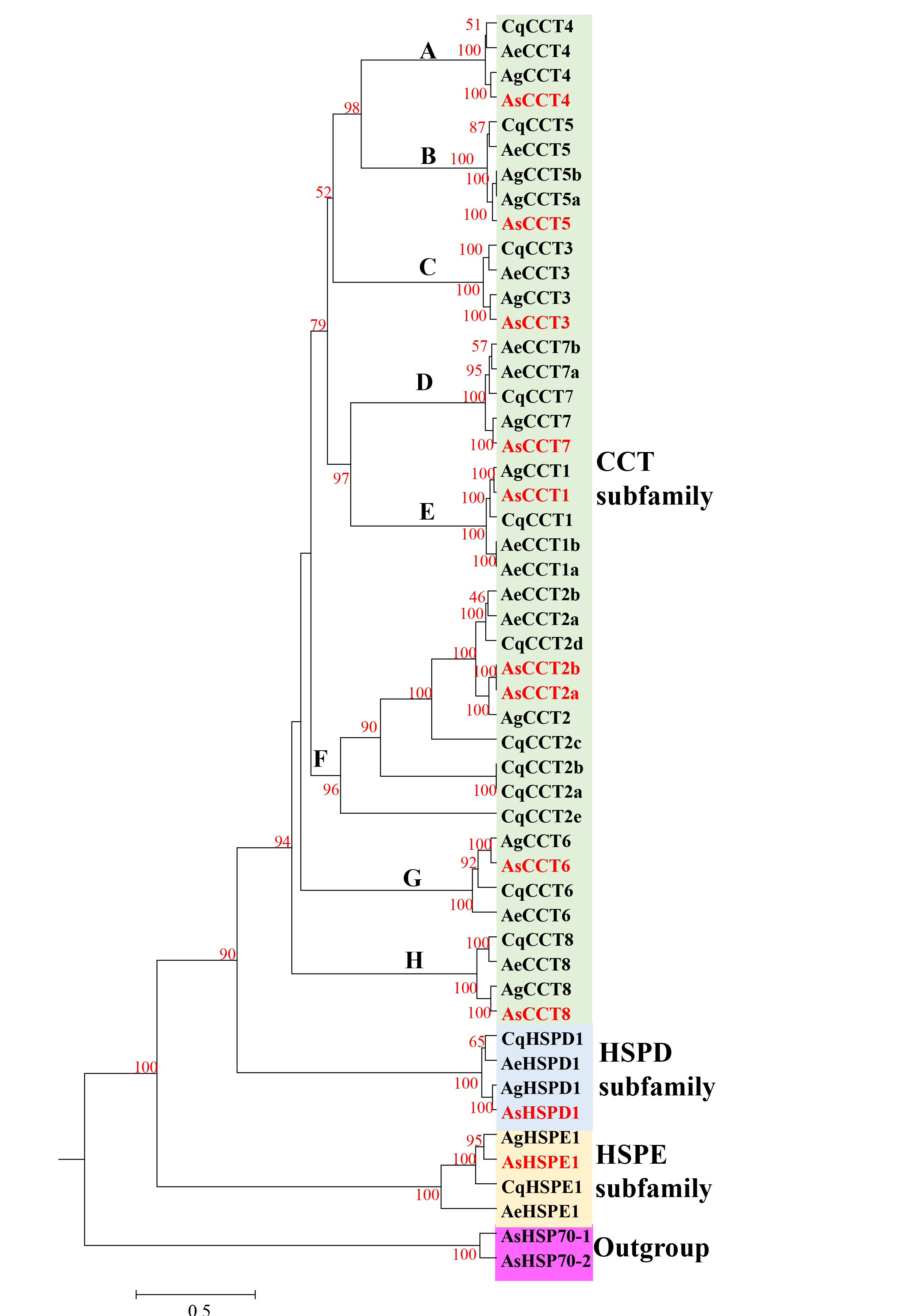

Supplement: Supplementary file 3 — Additional file 3: Fig. S2. Phylogenetic relationships of HSP genes from four insect species. The phylogenetic tree was constructed using the maximum-likelihood method based on predicted amino acid sequences. Analysis was performed with the program package MEGA5.0. The number at the branch point of the node represents the bootstrap values calculated from 1000 replications, and the gaps were deleted with pairwise deletion method. As, An. sinensis; Ag, An. gambiae; Cq, Cx. quinquefasciatus and Ae, Ae. aegypti. (A) HSP90 family; (B) HSP70 family; (C) Chaperonins family; (D) HSP40 family; (E) sHSP family. [file 12936_2019_2770_MOESM3_ESM.zip › Fig S2C.tif]

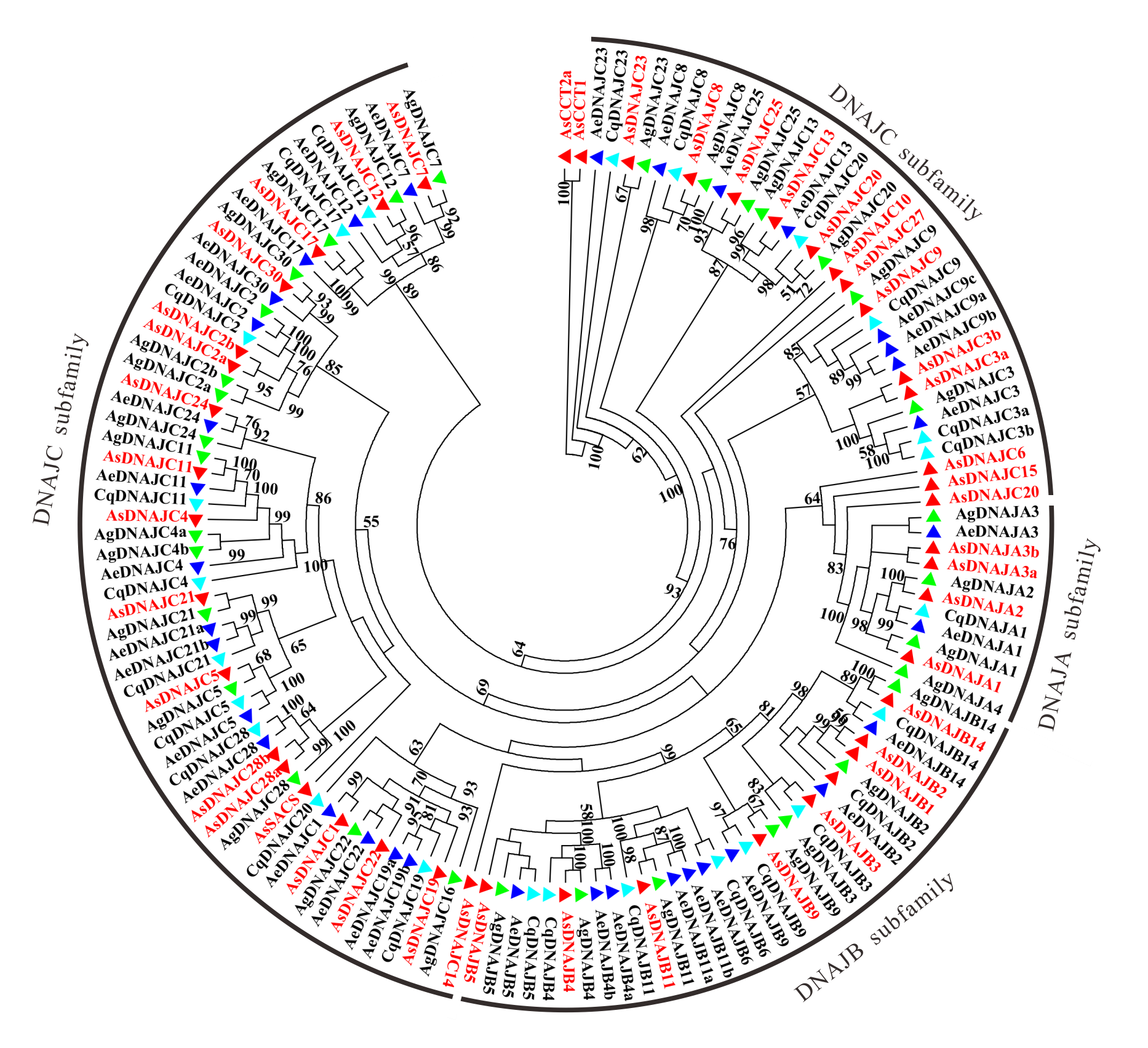

Supplement: Supplementary file 3 — Additional file 3: Fig. S2. Phylogenetic relationships of HSP genes from four insect species. The phylogenetic tree was constructed using the maximum-likelihood method based on predicted amino acid sequences. Analysis was performed with the program package MEGA5.0. The number at the branch point of the node represents the bootstrap values calculated from 1000 replications, and the gaps were deleted with pairwise deletion method. As, An. sinensis; Ag, An. gambiae; Cq, Cx. quinquefasciatus and Ae, Ae. aegypti. (A) HSP90 family; (B) HSP70 family; (C) Chaperonins family; (D) HSP40 family; (E) sHSP family. [file 12936_2019_2770_MOESM3_ESM.zip › Fig S2D.tif]

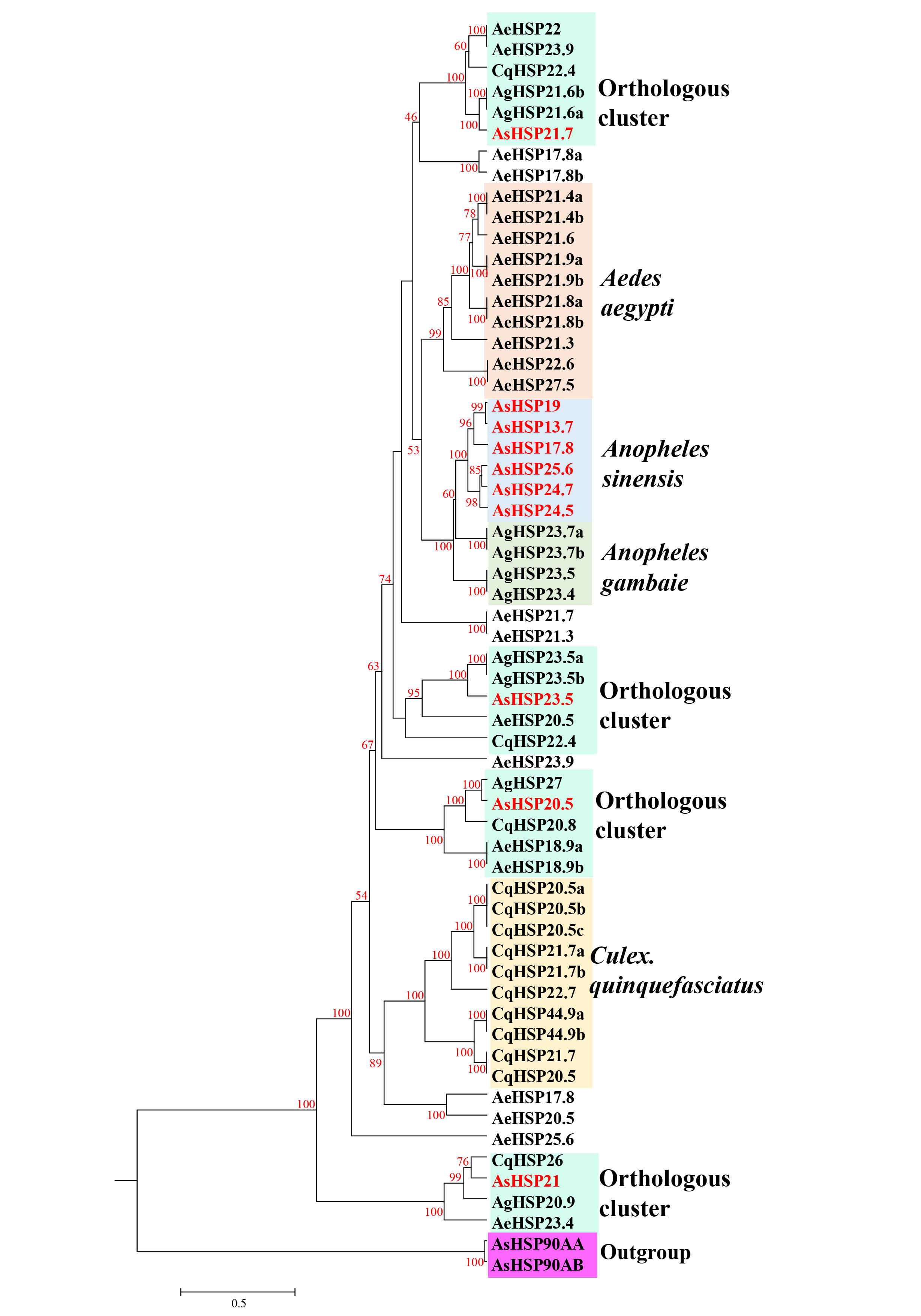

Supplement: Supplementary file 3 — Additional file 3: Fig. S2. Phylogenetic relationships of HSP genes from four insect species. The phylogenetic tree was constructed using the maximum-likelihood method based on predicted amino acid sequences. Analysis was performed with the program package MEGA5.0. The number at the branch point of the node represents the bootstrap values calculated from 1000 replications, and the gaps were deleted with pairwise deletion method. As, An. sinensis; Ag, An. gambiae; Cq, Cx. quinquefasciatus and Ae, Ae. aegypti. (A) HSP90 family; (B) HSP70 family; (C) Chaperonins family; (D) HSP40 family; (E) sHSP family. [file 12936_2019_2770_MOESM3_ESM.zip › Fig S2E.tif]
