# Supplementary material for: HSP superfamily of genes in the malaria vector Anopheles sinensis: diversity, phylogenetics and association with pyrethroid resistance
Source: Malar J. 2019 Apr 11;18:132. doi: 10.1186/s12936-019-2770-6 (PMC6460852; doi:10.1186/s12936-019-2770-6)
Supplement: Supplementary file 4 — Additional file 4: Table S2. Type and domain structure of 40 HSP40 (DNAJ) genes identified on An. sinensis genome. All these genes have complete open reading frame (ORF) sequences, with all supported by transcripts identified. [file 12936_2019_2770_MOESM4_ESM.doc]

**Table S2. Type and domain structure of 40 DNAJ(*HSP40*) genes identified on *An. sinensis* genome.** All these genes have complete open reading frame (ORF) sequences, with all supported by transcripts identified.

| **Subfamily/gene** | **Domain Structure** | **Accession ID** |
| --- | --- | --- |
| **DNAJA** |  |  |
| DNAJA1 | DnaJ-CXXCXGXG | ASI10013314 |
| DNAJA2 | DnaJ-CXXCXGXG | ASI10007271 |
| DNAJA3a | DnaJ-CXXCXGXG | ASI10000373 |
| DNAJA3b | DnaJ-CXXCXGXG | ASI10003362 |
| **DNAJB** |  |  |
| DNAJB1 | DnaJ-Transmembrane region | ASI10008434 |
| DNAJB2 | DnaJ-Transmembrane region | ASI10008436 |
| DNAJB3 | DnaJ | ASI10011791 |
| DNAJB4 | DnaJ-DnaJ_C | ASI10011296 |
| DNAJB5 | DnaJ-DnaJ_C | ASI10007539 |
| DNAJB9 | DnaJ-Thioredoxin-Transmembrane region | ASI10002400 |
| DNAJB11 | DnaJ-DnaJ_C | ASI10012810 |
| DNAJB14 | DnaJ-DUF1977 | ASI10008393 |
| **DNAJC** |  |  |
| DNAJC1 | DnaJ-C | ASI10010542 |
| DNAJC2a | DnaJ-SANT | ASI10000490 |
| DNAJC2b | DnaJ-RAC_head-SANT-SANT | ASI10001418 |
| DNAJC3a | 5*(TPR)-DnaJ | ASI10001314 |
| DNAJC3b | 5*(TPR)-DnaJ | ASI10000724 |
| DNAJC4 | DnaJ-Transmembran domain | ASI10012987 |
| DNAJC5 | DnaJ | ASI10003190 |
| DNAJC6 | S_TKc-PTEN_C2-DnaJ | ASI10016922 |
| DNAJC7 | DnaJ-Transmembran domain-SANT-SANT | ASI10002383 |
| DNAJC8 | DnaJ-coiled coil | ASI10002923 |
| DNAJC9 | DnaJ | ASI10002825 |
| DNAJC10 | DnaJ | ASI10006348 |
| DNAJC11 | DnaJ-DUF3395 | ASI10005126 |
| DNAJC12 | DnaJ | ASI10010542 |
| DNAJC13 | coiled coil-Pfam:DUF4339-DnaJ | ASI10010684 |
| DNAJC17 | DnaJ-RRM_1 | ASI10014935 |
| DNAJC18 | DnaJ | ASI10004326 |
| DNAJC19 | Transmembrane region-DnaJ | ASI10017171 |
| DNAJC20 | DnaJ-HSCB_C | ASI10004461 |
| DNAJC21 | DnaJ-Zn_U1-Znf_C2H2 | ASI10009501 |
| DNAJC22 | DnaJ-Jiv90 | ASI10017290 |
| DNAJC23 | DnaJ-Sec63 | ASI10006810 |
| DNAJC24 | DnaJ | ASI10011960 |
| DNAJC25 | Transmembrane domain-DnaJ-Transmembrane domain | ASI10013943 |
| DNAJC28a | DnaJ | ASI10000629 |
| DNAJC28b | DnaJ | ASI10001354 |
| DNAJ29 | SAP-DnaJ | ASI10014748 |
| DNAJC30 | DnaJ | ASI10017098 |
